# Expression of Iron Metabolism Genes Is Potentially Regulated by DOF Transcription Factors in *Dendrocalamus latiflorus* Leaves

**DOI:** 10.3390/ijms25158114

**Published:** 2024-07-25

**Authors:** Peng-Kai Zhu, Mei-Xia Lin, Mei-Yin Zeng, Yu Tang, Xin-Rui Li, Tian-You He, Yu-Shan Zheng, Ling-Yan Chen

**Affiliations:** 1 College of Landscape Architecture and Art, Fujian Agriculture and Forestry University, Fuzhou 350002, China; 2 College of Forestry, Fujian Agriculture and Forestry University, Fuzhou 350002, China

**Keywords:** DOF transcription factors, iron metabolism related genes, gene regulation, gene expression, *Dendrocalamus latiflorus*

## Abstract

Transcription factors (TFs) are crucial pre-transcriptional regulatory mechanisms that can modulate the expression of downstream genes by binding to their promoter regions. DOF (DNA binding with One Finger) proteins are a unique class of TFs with extensive roles in plant growth and development. Our previous research indicated that iron content varies among bamboo leaves of different colors. However, to our knowledge, genes related to iron metabolism pathways in bamboo species have not yet been studied. Therefore, in the current study, we identified iron metabolism related (IMR) genes in bamboo and determined the TFs that significantly influence them. Among these, DOFs were found to have widespread effects and potentially significant impacts on their expression. We identified specific DOF members in *Dendrocalamus latiflorus* with binding abilities through homology with *Arabidopsis* DOF proteins, and established connections between some of these members and IMR genes using RNA-seq data. Additionally, molecular docking confirmed the binding interactions between these DlDOFs and the DOF binding sites in the promoter regions of IMR genes. The co-expression relationship between the two gene sets was further validated using q-PCR experiments. This study paves the way for research into iron metabolism pathways in bamboo and lays the foundation for understanding the role of DOF TFs in *D. latiflorus*.

## 1. Introduction

Transcription factors (TFs) have a broad range of functions in gene regulation, influencing various physiological and developmental processes in plants [1,2,3]. Among these, the DOF (DNA binding with One Finger) TF family is unique to plants and plays critical biological roles [4]. Members of the DOF family contain a highly conserved DOF DNA-binding domain, consisting of 52 amino acids with a C2-C2 type zinc finger structure, allowing them to specifically bind to DNA sequences containing AAAG motif or its reversibly orientated motif CTTT [5,6,7]. Notably, DOF TFs have been found to regulate gene expression related to nutrient metabolism, stress responses, and developmental processes by binding to DNA sequences in target gene promoter regions [5,8,9,10,11,12]. This means that they have the potential to modulate the expression of target genes involved in iron uptake, transport, and homeostasis through the same mechanism, thereby influencing iron content. To date, no studies have identified or characterized DOF members in *Dendrocalamus latiflorus*.

Iron plays a crucial role in plant metabolism and is closely related to cellular processes, including photosynthesis, respiration, and DNA synthesis [13,14,15]. Its deficiency or excess can lead to significant metabolic disturbances, making its homeostasis vital for plant health and growth. *Bambusa multiplex* f. *silverstripe*, a clumping ornamental bamboo, is a natural chlorophyll-deficient form of *Bambusa multiplex* [16,17]. In the formal study, we discovered that the iron content varies among its differently colored leaves [18]. Additionally, since iron acts as a cofactor in the early stages of chlorophyll biosynthesis [19,20], it may cause thylakoid malformation and lead to changes in cell structure [21]. This led us to further explore the reasons behind this difference in iron accumulation and the regulatory mechanisms in bamboo leaves. However, there is currently no reference genome available for *B. multiplex*, which hinders our ability to understand the gene expression and regulatory mechanisms related to iron metabolism [22]. Fortunately, our laboratory has previously assembled a chromosome-level genome for *D. latiflorus*, which has the potential to become a model for clumping bamboo plants [23]. Additionally, *D. latiflorus*, a rapidly growing hexaploid bamboo, possesses a substantial biomass, giving it significant potential for carbon sequestration [24,25]. The expression of iron metabolism genes and the associated upstream regulatory mechanisms may mediate leaf development changes that could impact photosynthesis [20,26], ultimately affecting its carbon sequestration capacity. These conditions make it feasible to study iron metabolism at the genetic level and drive us to explore the molecular mechanisms of gene regulation involved in iron metabolism in bamboo leaves.

In this study, our aim was to elucidate the regulation of iron metabolism related (IMR) genes by DOF TFs in *D. latiflorus*. Firstly, we identified genes associated with iron metabolism in *D. latiflorus* and established the potential regulatory role of the DOF TF family in their expression. Subsequently, reliable regulatory relationships were confirmed through protein-DNA binding models, transcriptome data, and q-PCR experiments. This study provides new insights into the regulatory mechanisms of IMR genes in *D. latiflorus* and paves the way for understanding the role of DOF TFs in this context.

## 2. Results

### 2.1. IMR Genes in D. latiflorus

**Figure 1:**
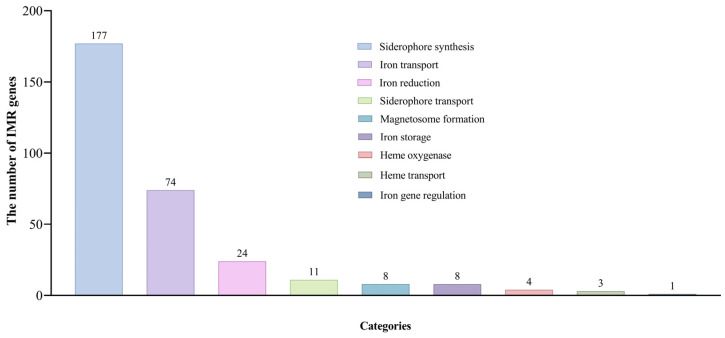
The number of IMR genes in different metabolic categories of *D. latiflorus*.

Plants have evolved efficient iron uptake mechanisms and sophisticated internal iron transport mechanisms, regulating gene expression in response to iron availability to maintain iron homeostasis [15]. In total, we identified 311 IMR genes in *D. latiflorus* (Figure 1; Table S1). The genes related to siderophore synthesis were the most numerous, totaling 177. This was followed by 74 genes related to iron transport. Additionally, there were 24 genes related to iron reduction and 11 genes associated with siderophore transport. The number of genes related to magnetosome formation and iron storage was the same, with 8 genes each. Furthermore, the detected genes related to heme oxygenase, heme transport, and iron gene regulation were fewer, numbering 4, 3, and 1, respectively.

### 2.2. TF Binding Sites (TFBS) in Promoter Regions of IMR Genes

**Figure 2:**
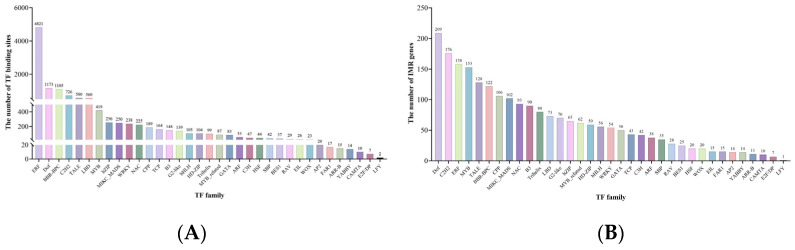
The number of TFBS from different TF families in IMR gene promoter regions (**A**), and the number of IMR genes in the promoter regions of each TF family that have binding sites (**B**).

In the promoter regions of IMR genes, we identified 11,857 TFBS belonging to 35 TF families (Figure 2A; Table S2). The ERF family had the highest number of binding sites, with 4821, followed by DOF and BBR-BPC, with 1173 and 1105 binding sites, respectively. These are the only three TF families with more than 1000 binding sites. The C2H2, TALE, and LBD families had between 500 and 1000 binding sites each, with 726, 580, and 560 binding sites, respectively. Additionally, 24 families had between 20 and 500 binding sites each. Only five families had fewer than 20 binding sites: FAR1, ARR-B, YABBY, CAMTA, E2F/DP, and LFY, with 17, 15, 14, 10, 7, and 2 binding sites, respectively.

Upon further examination, we found significant variability in the types and numbers of TFBS within the promoters of IMR genes. To identify the TF families that potentially have the broadest impact on IMR genes, we counted the number of IMR genes in each family’s promoter regions that have binding sites (Figure 2B). The results show that the DOF family has the highest number of IMR genes with binding sites in their promoter regions, with 209 genes, followed by the ERF family with 176 genes. Additionally, the ERF, MYB, TALE, BBR-BPC, CPP, and MIKC_MADS families each have over 100 genes, with 158, 153, 128, 122, 106, and 102 genes, respectively. Seven families have fewer than 20 IMR genes occupying their promoter regions, namely EIL, FAR1, AP2, YABBY, ARR-B, CAMTA, E2F/DP, and LFY, with LFY having the lowest impact with only 1 IMR gene in its promoter regions.

Overall, the ERF TF family have significantly more TFBS than any other family, with over four times the number of the second ranking DOF family. However, it occupies a smaller proportion of promoter region sequences compared to the DOF and C_2_H_2_ families. This suggests that ERF may exert a stronger influence on a smaller subset of genes. On the other hand, while the DOF family has relatively fewer TFBS compared to ERF, it occupies the largest proportion of promoter region sequences. We believe this indicates a broader regulatory role of the DOF family in IMR genes. Therefore, we will focus on analyzing this family in subsequent analyses.

### 2.3. DOF Members in D. latiflorus

**Figure 3:**
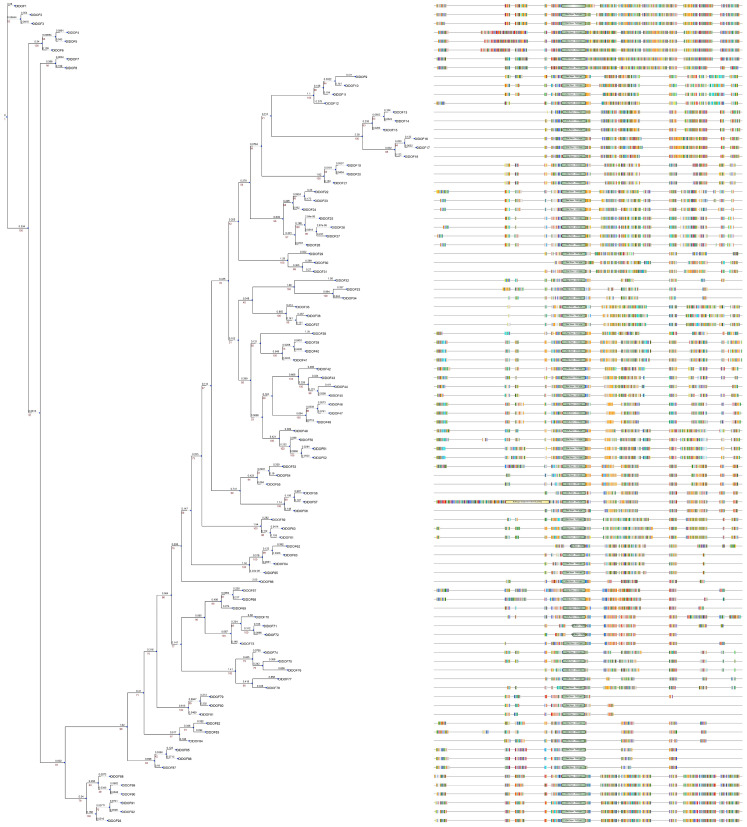
The phylogenetic relationship, conserved motifs, and multiple alignment diagram of 93 DlDOFs.

In the present study, we identified 33, 32, and 28 new DOF members in the A, B, and C genomes of *D. latiflorus*, respectively (Table S3). We renamed them according to their phylogenic relationship (Figure 3). The proteins encoded by these genes ranged in size from 143 to 554 amino acids, and the theoretical molecular weight of *D. latiflorus* DOF members (DlDOFs) varied from 14,956.73 to 60,809.6 Da, with isoelectric points (pI) ranging from 4.76 to 10.28. All DlDOF proteins were characterized as unstable and hydrophilic proteins. Signal peptide prediction analysis revealed that all DlDOF members contained signal peptide sequences. Transmembrane domain (TM) prediction showed that all DlDOFs contained one TM. Subcellular localization prediction indicated that DlDOFs are located in the nucleus, cytoplasm, mitochondria, and extracellular regions (including the cell wall). The majority of DlDOFs, 69 in total, are distributed in the nucleus. Additionally, 16 DlDOFs are located in the cytoplasm, 6 in the mitochondria, and only 2 are found in the extracellular regions.

### 2.4. Potential DOFs Regulating IMR Genes

The predictions of TFBS suggest that numerous DOFs might influence the expression of IMR genes. To further identify potential DOFs regulating IMR gene expression, we first identified the conserved domains of DOF binding sites. The results showed that these sequences contain two conserved domains, referred to as Motif1 and Motif2, which are present in the promoter regions of 95 and 126 IMR genes, respectively (Table S4). This indicates that DlDOFs capable of binding to these motifs in the promoter regions might regulate the expression of the corresponding IMR genes. Moreover, searches of these motif sequences in the JASPAR database revealed that in *Arabidopsis*, 14 DOFs can bind to both motifs, 14 DOFs can only bind to Motif1, and 23 DOFs can only bind to Motif2 (Table S5). The protein-DNA interactions of these AtDOFs have been validated through DAP-Seq experiments [27], making them highly reliable. To identify DOFs in *D. latiflorus* capable of binding to these motifs, we compared them with these AtDOFs based on sequence similarity. We identified 53 DlDOFs homologous to these genes (Table S6). Subsequently, these DlDOFs were linked to IMR genes containing Motif1 or Motif2, resulting in the identification of 7862 potential interactions (Table S7). These DlDOFs might regulate IMR gene expression by binding to their promoters.

### 2.5. Expression of DlDOFs and IMR Genes in D. latiflorus Leaves

**Figure 4:**
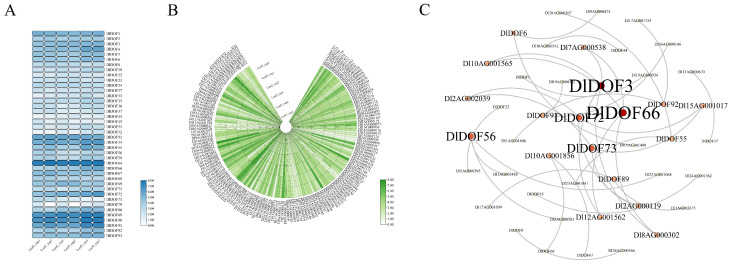
Transcriptome analysis of genes in *D. latiflorus* leaves. Expression heatmap of DlDOFs in leaves (**A**). Expression heatmap of IMR genes in leaves (**B**). Co-expression network of DlDOFs and IMR genes (**C**). Leaf1, Leaf2, and Leaf3 refer to mature leaves collected from three different *D. latiflorus* cutting seedlings.

We examined the expression of DlDOFs and IMR genes in *D. latiflorus* leaves. The results indicated that 39 out of 93 DlDOFs had an average Transcripts Per Million (TPM) value greater than 1 across all leaf samples (Figure 4A), while 252 out of 272 IMR genes had an average TPM value greater than 1 (Figure 4B). To delve deeper into the impact of DOFs on IMR gene expression, we generated a co-expression network between the two gene sets using a threshold of Pearson’s Correlation Coefficients (PCCs) > 0.9 (Figure 4C). We identified 36 connections between the two gene sets, involving 18 DlDOFs and 27 IMR genes. Among these, *DlDOF3* and *DlDOF66* had the most connections with IMR genes, each regulating the expression of four IMR genes. Following them, *DlDOF56*, *DlDOF7*, and *DlDOF73* each had three connections with IMR genes. Five DlDOFs had two connections with IMR genes: *DlDOF55*, *DlDOF6*, *DlDOF89*, *DlDOF91*, and *DlDOF92*. Lastly, eight DlDOFs had only one connection with IMR genes.

### 2.6. Molecular Docking of Key DlDOFs and Binding Sites in IMR Gene Promoters

**Figure 5:**
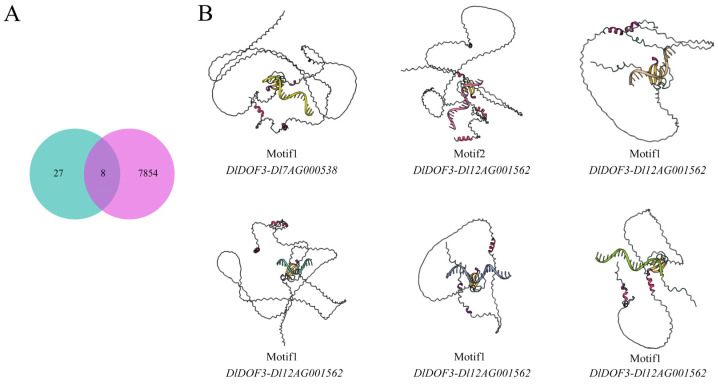
Key DlDOFs; selection and molecular docking. Venn diagram illustrating co-expression and potential regulatory relationships between DlDOFs and IMR genes through promoter binding (**A**). Green circles represent the number of co-expression relationships, while magenta circles represent potential regulatory relationships. Protein-DNA interaction models (**B**). Text below the model indicates the motif types present in TFBS and the binding of DlDOFs to IMR gene promoters.

Despite predicting 7862 potential regulatory relationships between DlDOFs and IMR genes based on data, transcriptome data only supported 35 co-expression relationships between the two gene sets. To determine reliable gene interactions and select key DlDOFs, we examined the overlapping relationships between the two datasets (Figure 5A). The results revealed that only eight relationships were present in both datasets, involving six DlDOFs (*DlDOF3*, *DlDOF37*, *DlDOF55*, *DlDOF56*, *DlDOF6*, *DlDOF8*) and eight IMR genes (*Dl7AG000538*, *Dl12AG001562*, *Dl13AG000670*, *Dl23AG001841*, *Dl3AG000581*, *Dl23AG001068*, *Dl15AG001499*, *Dl28AG000366*). Except for two genes, *DlDOF3* and *DlDOF56*, which each had connections with two IMR genes, the other DlDOFs were linked to only one IMR gene. This suggests that *DlDOF3* and *DlDOF56* may play relatively important roles in regulating IMR gene expression. Subsequently, we performed molecular docking calculations for six intersecting DlDOFs with the DOF binding sites in the promoter regions of the target IMR genes (Table S8).

Typically, lower binding energies indicate stronger binding interactions. The docking results showed that among the interactions between three pairs of DlDOFs and the binding sites in the promoter regions of IMR genes, six interactions had binding energies less than −4 kcal/mol, and three had binding energies between −3 and −4 kcal/mol. The binding energy between *DlDOF56* and the binding sites in the promoter of the *Dl23AG001068* gene was the lowest at −5.8 kcal/mol, followed by *DlDOF3*-*Dl7AG000538* at −4.9 kcal/mol. Additionally, the strongest interactions were found between *DlDOF3*-*Dl7AG000538*, *DlDOF3*-*Dl12AG001562*, and *DlDOF56*-*Dl23AG001068* (Figure 5B). The promoter regions of *Dl12AG001562* and *Dl23AG001068* each contained three DOF binding sites, indicating that they are highly influenced by DlDOFs. However, the binding energies of *DlDOF6*-*Dl15AG001499* and *DlDOF56*-*Dl3AG000581* were −3.7 and −3.5 kcal/mol, respectively, suggesting weaker interactions. Notably, their DOF binding sites both contained Motif1. Coincidentally, we found that among the entries containing Motif1, only *DlDOF3*-*Dl7AG000538* had a binding energy less than −4 kcal/mol, indicating that DlDOFs may have weaker binding interactions with sites containing Motif1 compared to sites containing Motif2. Further, the binding energy between *DlDOF55* and the TFBS of *Dl23AG001841*, which contains Motif1, reached as high as 12.9 kcal/mol, suggesting a weak interaction. Therefore, the previously observed co-expression relationship between these two genes might be coincidental or influenced by other genes.

### 2.7. q-PCR Validation

**Figure 6:**
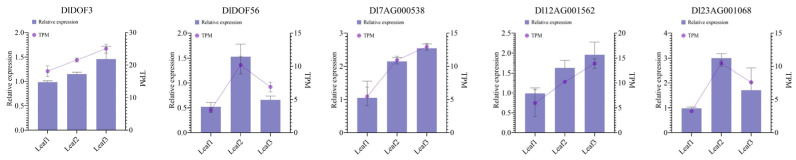
Relative expression patterns of two DlDOFs and three IMR genes in leaves.

Using q-PCR, we determined the relative expression levels of two DlDOFs and three IMR genes potentially regulated by them in leaf samples identical to those used in RNA-seq (Figure 6; Table S9). Results indicated that all five genes had the lowest expression in sample Leaf1. *DlDOF3*, *Dl7AG000538*, and *Dl12AG001562* exhibited the highest expression in sample Leaf3, while *DlDOF56* and *Dl23AG001068* showed the highest expression in sample Leaf2. Furthermore, correlation analysis of the relative expression patterns of these five genes supported high correlations between *DlDOF3*-*Dl7AG000538*, *DlDOF3*-*Dl12AG001562*, and *DlDOF56*-*Dl23AG001068* (Figure S1). We also compared the fold changes in relative expression and TPM values between different samples (Figure S2), using sample Leaf1 as a control, which demonstrated a strong correlation between q-PCR and transcriptome data, validating the reliability of our sequencing data.

## 3. Discussion

Transcriptional regulation is a key process in defining cell characteristics, growth, differentiation, and development [1,28]. As an important pre-transcriptional regulatory mechanism, TFs can regulate the expression of target genes by binding to specific DNA sequences [29,30]. However, this binding is influenced by various factors. For example, chromatin accessibility determines whether specific regions can be bound by DNA-binding proteins [31,32], and this accessibility is constrained by many epigenetic conditions. For instance, cytosine methylation at the fifth carbon (5mC) is a modification present in the DNA of all known vertebrates and terrestrial plants [33,34] which plays a crucial role in genome defense [35], and can influence chromatin accessibility [36,37]. Additionally, DNA is typically wrapped around chromatin nucleosomes. Changes in their chromatin structure result in pleiotropic developmental phenotypes [38,39], impacting the growth and development of plants [40,41], and playing a crucial role in their adaptation to environmental stresses [42,43]. This implies that chromatin structure or DNA methylation can affect chromatin accessibility, thereby regulating the binding of TFs and subsequently influencing downstream gene expression. This might explain why we predicted a large number of potential DOF TFs regulating IMR genes in this study, yet only a few were supported by transcriptional level data in bamboo leaves (Figure 5A). Based on these reasons, we speculate that more relationships between the two gene groups might be observed in other tissues of bamboo. Overall, the current research on the role of TFs in bamboo is still in its early stages. To gain deeper insights into the functions of DOF family members, future research could explore strategies focusing on TF binding influenced by epigenetic factors. This may be an important driver for bamboo and other ecological species to quickly adapt to evolution through self-regulation of transcriptional levels.

For TFs themselves, their binding affinity is influenced by protein structure and various interfering factors such as enzymes [44], metal ions [45], and hormones [46]. Although molecular docking calculations indicate that some DOF TFs have strong binding affinity to the IMR gene promoter sites, the number is much lower than expected (Table S8). Current research on plant protein structures is limited and mainly focused on model species [47,48]. In the protein sequences of DlDOF family members, aside from the docking sites in the central region, most residues do not show reliable protein structure predictions (Figure 5B). This may result in some DOF TFs exhibiting weak binding affinity to the binding site sequences in molecular docking. Furthermore, while there are currently no reports of other DOF TFs interacting with metal ions or enzymes to cause changes in their activity, the limited understanding of the DlDOF protein structure suggests potential interactions with these substances. Additionally, DOF, as a broadly acting family of TFs [5], suggests that more DOF proteins are expected to exhibit stronger binding capabilities to target sequences. However, this speculation currently lacks empirical evidence to support it. Observing the structure of DlDOF through cryo-electron microscopy represents an optimal approach for directly targeting protein studies [49], but these studies are costly and constrained by researchers’ economic conditions. Therefore, to further explore the molecular functions of DOF TFs, our laboratory plans to proceed with testing antibodies suitable for DlDOFs and using CHI-Seq and DAP-seq to specifically determine the binding sequences of DOF proteins across the entire bamboo genome.

In previous studies, we observed higher iron content in chlorotic bamboo leaves [18]. Building on these findings, in the current study, we investigated the potential regulatory role of DOF TFs on IMR genes. We believe that knocking out or overexpressing some DlDOF members will affect the expression of IMR genes, and due to the broad role of TFs [1], it may have more significant effects on plant cells. However, the specific impact of IMR protein abundance on iron metabolism is currently unclear. Both heme and chlorophyll synthesis depend on the common precursor protoporphyrin [50], which have antagonistic effects on each other [51]. The genes in the tetrapyrrole synthesis pathway are downregulated in chlorotic bamboo leaves [18], indicating reduced accumulation of protoporphyrin, which may lead to decreased synthesis of iron-containing heme and chlorophyll. However, it is still unclear whether the decrease in iron content is due to reduced protoporphyrin levels leading to the inability to synthesize and accumulate iron-containing heme, or directly due to changes in cell structure caused by chlorophyll deficiency, resulting in decreased IMR gene expression and thus affecting the iron metabolism pathway. Therefore, we believe that the next step in studying the function of the IMR gene should further explore the relationship between iron metabolism and other related biological pathways from both physiological and genetic perspectives.

## 4. Materials and Methods

### 4.1. Plant Materials, RNA-Seq, Sequencing Reads Alignment and Quantification

The samples were collected from the Bamboo Botanical Garden of Fujian Agriculture and Forestry University, located in Cangshan District, Fuzhou City, Fujian Province, China (26°05′ N, 119°14′ E). The plant experiments and field studies conducted in this study, including the collection of plant material, comply with relevant institutional, national, and international guidelines and legislation. We collected fresh leaves from three *D. latiflorus* cutting seedlings, each propagated from stems cut from different 20-year-old bamboo plants. Each cutting seedling was over one year old, with stems exceeding 1 m in height. These seedlings were grown under partial shade in a forest setting without any artificial light control. We randomly selected three of these seedlings and collected mature leaves from each. In this study, we labeled the mature leaves collected from the first, second, and third seedlings as Leaf1, Leaf2, and Leaf3, respectively. Before collecting the leaves, alcohol was uniformly sprayed on both sides of the leaves to ensure complete surface coverage. The alcohol was allowed to act on the leaf surfaces for approximately 30 s to 1 min for thorough disinfection. Subsequently, the alcohol sprayed leaves were gently placed in deionized water to remove any residual alcohol and potential dead microorganisms from the surface, for a duration of about 2 min. The collected leaves were promptly mixed and then flash frozen in liquid nitrogen. Subsequently, they were stored at −80 °C. Total RNA from the leaves was extracted using the RNA prep Pure Plant Kit (Tiangen, Beijing, China). The quality of total RNA was assessed using 2% agarose gel electrophoresis. RNA concentration was measured using a NanoPhotometer^®^ spectrophotometer (IMPLEN, Westlake Village, CA, USA) and a Qubit^®^ RNA assay kit with a Qubit^®^ 2.0 fluorometer (Life Technologies, Carlsbad, CA, USA). RNA integrity was evaluated using an RNA Nano 6000 assay kit on an Agilent^®^ Bioanalyzer 2100 system (Agilent Technologies, Santa Clara, CA, USA), setting a benchmark RNA integrity number of 7 as the standard for quality assessment. Library construction was carried out using the NEB-Next^®^ Ultra^™^ RNA library prep kit for Illumina^®^ (NEB, Ipswich, MA, USA). All samples were sequenced on the Illumina^®^ 6000 platform, generating 150 bp paired-end reads.

We performed the initial processing of raw RNA-Seq reads using fastp v0.23.2 [24], removing sequences with adapters and sequences where more than 50% of the total length had bases with a Qphred ≤ 20, as well as sequences with an N-base percentage greater than 15%. The remaining clean reads were aligned to the reference genome using STAR v2.7.8a [52] and quantified using featureCounts v2.0.1 [53]. Then TPM values were calculated using a custom python script. log_2_(TPM + 1) was used to generate a heat map using TBtools v2.097 [54].

### 4.2. Identification of IMR Genes and DOFs in D. latiflorus

The haplotype genome of *D. latiflorus* were downloaded from BambooBase [55]. We selected the longest transcript as the representative transcript for each gene and generated CDS and translated protein sequences using SeqKit v0.15.0 [56]. Based on these protein sequences, the FeGenie v1.2 [57] was employed to identify IMR genes in *D. latiflorus*. DOF members (DOFs) in *D. latiflorus* were initially identified DOFs from *Arabidopsis thaliana* and *Oryza sativa* genome-wide using blastp function in Blast v2.10.1 [58]. Additionally, *D. latiflorus* protein sequences were screened using the DOF domain model (PF02701) from the Pfam database [59] via the hmmsearch function of HMMER v3.3.2 [60] with *p*-value < 1 × 10^−5^. Subsequently, the search results were used with the hmmbuild function to construct a species-specific hidden Markov model. The newly generated model was then used to re-identify DlDOFs using hmmersearch with *p*-value < 1 × 10^−20^. The union of results from both methods was examined using Interproscan v5.63-95.0 [61] to verify the conserved domains of DOF and remove sequences lacking the conserved DOF domain, resulting in the reliable identification of DlDOFs. We used the ProtParam tool on the ExPASy platform [62] for protein physicochemical property analysis, and the WoLF PSORT server [63] for predicting subcellular localization.

### 4.3. Mutiple Alignment and Phylogenic Analysis

To investigate the phylogenetic relationship of DlDOFs, we aligned these genes using Muscle v5.1 [64] and trimmed the alignment result automatically using trimAL v1.4 [65]. We constructed a maximum likelihood (ML) tree using IQ-TREE v2.1.2 [66] with the MFP model and 1000 times bootstrap replicates and visualized the phylogenetic tree and alignment result using custom python script with ETE v3.1.3 toolkit [67].

### 4.4. Identifying TF Binding Sites in IMR Gene Promoters and Predicting Potential Regulatory TFs

We used the 1500 bp upstream region from the transcription start site of IMR genes as the promoter region. The DOF binding sites in this promoter region were scanned using the PlantRegMap server [68]. Next, the MEME suite [69] was used to identify potential DOFs that regulate the expression of IMR genes. Specifically, sequences of all potential binding sites were scanned with the meme function, setting the parameter to the number of motifs 3 to identify the conserved domains of these sequences. Finally, these domain sequences were searched using the TOMTOM function in the JASPAR non-redundant database [70] to find the *Arabidopsis* DOF genes (AtDOFs) that bind to these domains.

### 4.5. Key DlDOFs Selection and Molecular Docking

To identify the DOF TFs in *D. latiflorus* that potentially regulate the expression of IMR genes, we first obtained AtDOFs from the JASPAR database [70] with experimental evidence supporting their binding to the promoter regions of target genes. Subsequently, based on Blastp sequence similarity results with thresholds set at bit score >90, *p*-value < 1 × 10^−30^, and identity >75%, we identified homologous proteins of these AtDOFs in *D. latiflorus*. Additionally, using GCEN v0.6.3 [71], we applied a PCCs threshold of ≥0.9 between TPM values to identify DlDOFs that co-express with IMR genes. The intersection of these two sets of genes was taken to obtain DlDOFs that are most likely to regulate the expression of IMR genes in *D. latiflorus* by binding to their promoter regions.

We used AlphaFold 3 server [72] to construct protein-protein and protein-DNA interaction models, Avogadro v1.2.0 [73] to build DNA models, and AutoDock Tools v1.5.7 [74] for receptor and ligand preprocessing, including desolation and hydrogenation. Protein binding sites were identified using the DeepSite server [75]. For proteins exceeding 1000 residues, binding sites were manually determined. AutoDock Vina v1.1.2 [76] was employed to compute binding energies.

### 4.6. q-PCR Validation

Collected leaves used for transcriptome sequencing were placed into a mortar and ground with liquid nitrogen. Total RNA was isolated from the ground material using the RNA prep Pure Plant Kit (Tiangen, Beijing, China), and then reverse transcribed into cDNA using PrimeScript^™^ RT reagent Kit (Perfect Real Time, Takara, Japan). q-PCR analysis of selected genes was performed Applied Biosystems^®^ 7500 Real-Time System (Applied Biosystems, Foster City, CA, USA) using Hieff^®^ qPCR SYBR Green Master Mix (Low Rox Plus) kit (Yisheng, Shanghai, China). The q-PCR amplification program was: 95 °C pre-denaturation for 5 min, 95 °C denaturation for 10 s, 60 °C annealing for 20 s; 72 °C extension for 20 s, 40 cycles. GAPDH was used as an internal reference gene [77], and the relative expression of each gene was calculated using the 2^−ΔΔCT^ method. The primers used in this study are shown in Table S10.

## Data Availability

The data supporting the findings of this work are available within the Supplementary Materials. Please request further information from the corresponding author.

## Supplementary Materials

The following supporting information can be downloaded at: https://www.mdpi.com/article/10.3390/ijms25158114/s1.
