# Supplementary material for: Expression of Iron Metabolism Genes Is Potentially Regulated by DOF Transcription Factors in Dendrocalamus latiflorus Leaves
Source: Int J Mol Sci. 2024 Jul 25;25(15):8114. doi: 10.3390/ijms25158114 (PMC11311721; doi:10.3390/ijms25158114)
Supplement: Supplementary file 1 [file ijms-25-08114-s001.zip › Figure S1-S2.pdf]

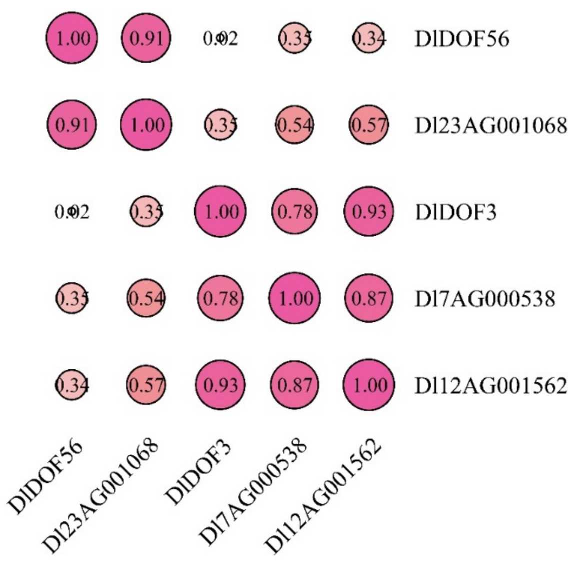

Figure S1. The relative expression correlation of two DlDOFs and three IMR genes in leaves.

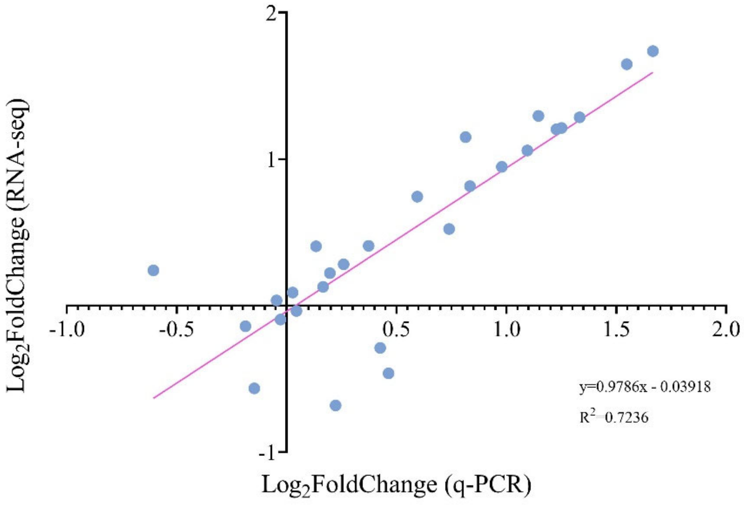

Figure S2. The linear regression between transcriptome data and q-PCR data.
